# *Ficus carica* leaf extract ameliorates cardiac injury through Nrf2/Keap1 pathway activation and dual oxidase inhibition

**DOI:** 10.22038/ijbms.2025.88664.19148

**Published:** 2025

**Authors:** Najeeb Ullah Khan, Shamshad Ul Hassan, Bilal Aslam, Saqib Umer

**Affiliations:** 1 Institute of Physiology and Pharmacology, University of Agriculture, Faisalabad, Pakistan; 2 Department of Theriogenology, University of Agriculture, Faisalabad, Pakistan

**Keywords:** Anti-oxidants, Cardio protection, High-fat diet, Isoproterenol, Myocardial infarction, Oxidative stress, Phytomedicine

## Abstract

**Objective(s):**

To investigate the therapeutic potential of *Ficus carica *leaf extract (FCLE) against high-fat diet (HFD) coupled with isoproterenol-induced cardiac injury in a rat model that mimics myocardial infarction.

**Materials and Methods:**

HPLC was performed to check the phytochemical composition of FCLE. Analysis of the drug-likeness of phytochemicals and molecular docking was conducted. Four groups of rats were allocated as negative control (NC), positive control (PC), standard (STD), and FCLE treatment groups. After the experiment, serum samples were collected to carry out biochemical analyses. Histopathological assessments of the heart and aorta tissues were performed. The heart tissue gene expression analysis was conducted.

**Results:**

***:*** Four active compounds were identified in HPLC. Drug-likeness analysis of bioactive phytochemical compounds from FCLE indicated no violations of Lipinski’s and Veber’s rules, except for one compound. Quercetin and chlorogenic acid exhibited high affinity for Duox1 and Keap1 (<-8 kcal/mol). FCLE demonstrated a significant reduction in Troponin I (*P*<0.01), CK-MB (*P*<0.001), triglycerides (*P*<0.001), total cholesterol (*P*<0.001), LDL-C (*P*<0.001), MDA (*P*<0.001), and NO (*P*<0.0001) alongside significant increases in HDL-C (*P*<0.01), SOD (*P*<0.001), and CAT (*P*<0.0001) when compared to PC. FCLE treatment significantly (*P*<0.0001) down-regulated gene expressions of Duox1, Duoxa1, Duoxa2, Bax, and Bad, whereas the expressions of Nfe2l2, Nrf1, and Bcl2 were significantly (*P*<0.0001) up-regulated when compared with PC.

**Conclusion:**

Our results suggest that FCLE mitigates cardiac injury by modulating oxidative stress and apoptosis through dual oxidases, the Nrf2/Keap1 pathway, and related apoptotic signaling cascades.

## Introduction

Myocardial Infarction (MI) is a form of cardiac injury caused by prolonged ischemia of cardiac tissue due to an inadequate oxygen supply through the coronary artery, and it is recognized as the most lethal cardiovascular disease (1). The World Health Organization predicted that global annual deaths from cardiovascular diseases (CVDs) will rise from 18.1 million in 2010 to a staggering 24.2 million by 2030 (2). The incidence of MI is 1,655 per 100,000 individuals, affecting 1.72% of the global population (3). MI is linked to apoptosis, inflammation, and oxidative stress, resulting in significant loss of heart muscle and impaired left ventricular function (4). The risk factors implicated in the pathogenesis of MI include hypercholesterolemia, hypertension, diabetes mellitus, and smoking, irrespective of age and sex (5). Mechanistically, these factors contribute to the production of reactive oxygen species (ROS), increased inflammation, and cardiomyocyte necrosis (6).

NADPH oxidases represent a family of flavoenzymes that include several isoforms, specifically NOX1-5, and dual oxidases 1 and 2 (DUOX1-2), which directly contribute to ROS generation (7, 8). Under normal conditions, a balance between ROS and anti-oxidants exists; however, excessive ROS can lead to various CVDs, including MI (9). During oxidative stress, the Nrf2 pathway acts as a key regulator of anti-oxidant defenses following MI. This activation decreases oxidative stress, reduces apoptosis, suppresses inflammation, and prevents pathological remodeling of the heart (10).

Isoproterenol (ISO) is a synthetic catecholamine and β-adrenergic receptor agonist, which induces irreversible injury to myocardial cells and ultimately MI in rats through subcutaneous injection (11). The resulting electrical and blood flow alterations in the heart closely resemble those observed in human MI, making this rat model valuable for testing potential cardioprotective medications (12). Most commonly used drugs for the procurement of MI, such as angiotensin-converting enzyme inhibitors and beta-blockers, provide benefits, but their limitations drive the search for alternative therapies. Natural products have played a significant role in developing treatments for CVDs, underscoring their potential in this field (13).

*Ficus carica,* commonly known as fig, belongs to the *Moraceae* family and is among the largest genera of medicinal plants, holding great promise for the future of natural medicine (14, 15). Multiple phenolic compounds are found in* F. carica,* including caffeic acid, chlorogenic acid, quercetin, ferulic acid, syringic acid, quinol, catechin, and gallic acid (16). These molecules exhibit a high anti-oxidant potential and offer possible beneficial effects through a synergistic action (17).

Our study aims to investigate the therapeutic potential of FCLE against high-fat diet (HFD) coupled with isoproterenol-induced cardiac injury/MI in the rat model by employing an integrated approach of analysis of the drug-likeness of phytochemicals, molecular docking, and in vivo evaluation of the anti-oxidant (Nrf2/Keap1) pathway, along with relevant apoptotic signaling, to assess FCLE’s ability to mitigate oxidative stress and cardiac damage.

## Materials and Methods

### Reagents, drugs, and chemicals

Isoproterenol hydrochloride (Sigma-Aldrich, Catalog Number: I5627) was used to induce MI. Metoprolol tartrate (Merol, manufactured by Atco, purchased from a local pharmacy in Faisalabad, Pakistan) was used as the standard treatment. All other reagents and chemicals used in this experiment were purchased from certified suppliers.

### Collection and preparation of leaf extract

Fresh *F. Carica *leaves were obtained from healthy, mature trees. The leaves were authenticated at the Herbarium of the Department of Botany, and a voucher specimen (voucher no. 1017-3-23) was archived at the Herbarium. The collected leaves were rinsed with distilled water to eliminate surface contaminants, then dried at room temperature (25 ± 2 °C) under shade with adequate ventilation until completely dried and ground into a fine powder using an electric grinder. The powdered leaves were extracted by maceration with 70% ethanol at room temperature for 72 hr with intermittent shaking, and the resulting extract was filtered using the Whatman No. 1 filter paper. The filtrate was then concentrated using a rotary evaporator to yield a crude extract, which was then stored at -20 °C until further experimentation. The extract was subjected to HPLC evaluation, phytochemical analysis, and anti-oxidant activity (18).

### Evaluation of FCLE phytochemical analysis and anti-oxidant activity

The phytochemical analysis of FCLE includes total phenolic content (TPC), which was evaluated by using the Folin-Ciocalteu method, and total flavonoid content was measured using a colorimetric method (19). The anti-oxidant activity of FCLE was assessed by the 2,2-diphenyl-1-picrylhydrazyl (DPPH) assay and ferric reducing anti-oxidant power (FRAP) assay (19, 20).

### Analysis of drug-likeness of FCLE phytochemicals

The physicochemical characteristics of FCLE bioactive compounds were sourced from the PubChem database (21) and the SwissADME web server (22). The drug-likeness of FCLE phytochemicals was assessed by Lipinski’s rule (23) and Veber’s rule (24).

### Molecular docking

Molecular docking was performed to determine the docking score/binding affinities of FCLE bioactive compounds identified through HPLC with their target receptors. The molecular structures of target receptors were retrieved from the RCSB protein database (http://www.rcsb.org/), and the PubChem databases were used to retrieve the structure of bioactive phytochemical compounds (21). The target receptors were prepared for molecular docking by using Chimera. To dock the receptors with ligands, PyRx software was utilized, and the optimal structure was selected based on the lowest binding energy. Docking results were visualized using Discovery Studio software.

### Ethical approval

All experimental procedures were conducted following the guidelines outlined in the National Biosafety Rules 2005, Punjab Biosafety Rules 2014, Punjab Animal Health Act 2019, and the Bioethics protocol, and were approved by the Institutional Biosafety and Bioethics Committee (Approval number: 1547/ORIC).

### Experimental design

Thirty-two male Wistar rats, each weighing 200 ± 20 g, were obtained and maintained under standard laboratory conditions at the animal house, adhering to a 12-hour light/dark schedule, with controlled temperature (26 ± 2°C) and humidity (40–60%). Following a 7-day acclimatization period, rats were randomly assigned to four groups (n=8 per group). Four groups of rats were allocated as negative control (NC) that received a standard chow diet, positive control (PC) received an HFD and ISO, standard (STD) received an HFD and ISO along with standard cardioprotective drug Metoprolol, and the FCLE treatment group received *F. Carica* leaf extract along with an HFD and ISO, as shown in Table 1. 

The HFD was prepared by mixing 350 g of hydrogenated vegetable ghee with 650 g of basic chow diet per kilogram of feed. The chow diet was composed of maize, soybean meal, full-fat soybean, wheat offal, bone meal, lysine, methionine, and salt (25). According to the manufacturer’s specifications, Dalda ghee contains saturated fat, monounsaturated fat, polyunsaturated fat, and trans-fat per 100 g. The composition of the basic chow diet and ghee is depicted in Table 2. 

The HFD was fed for 14 days, and ISO was administered subcutaneously at a dose rate of 30 mg/Kg/day on 13 and 14 days (26, 27). Metoprolol was administered orally at a dose rate of 20 mg/Kg/day from day 14 to 21. FCLE was administered orally at the dose rate of 300 mg/Kg/day from day 14 to 21. After the 21-day experimental period, all rats were euthanized according to approved ethical procedures. The serum samples were collected to perform biochemical analyses. Heart and aorta tissue samples were fixed in 10% neutral buffered formalin for histological analysis. The sections of the left ventricle were rapidly dissected, snap-frozen in liquid nitrogen, and stored at -20 °C in TRIzol reagent for RNA extraction and subsequent gene expression analysis.

### Biochemical analysis

Serum levels of cardiac biomarkers (Troponin I and CK-MB), lipid profile components (total cholesterol, triglycerides, HDL-C, and LDL-C), and oxidative stress markers (SOD, CAT, MDA, and NO) were determined using commercially available enzyme-linked immunosorbent assay (ELISA) kits as per the manufacturer’s guidelines.

### Gene expression analysis (qRT-PCR analysis)

Total RNA extraction from snap-frozen left ventricular tissue was executed using the TRIzol method according to the manufacturer’s instructions (Ambion TRIzol® by Thermo Scientific™, Massachusetts, USA; Catalogue No: 15596026), and isolated RNA was quantified by using the NanoDrop® ND-1000 Spectrophotometer. The extracted mRNA underwent reverse transcription into complementary DNA (cDNA) by following the manufacturer’s protocol through the RevertAid First Strand cDNA Synthesis Kit (Thermo Scientific™, Massachusetts, USA; Catalogue No: K1622). Quantitative real-time PCR (qRT-PCR) was employed for relative quantification of mRNA gene levels, using Maxima SYBR Green/ROX qPCR Master Mix (Thermo Scientific™, Massachusetts, USA; Catalogue No: K0221). The primer sequence and amplified PCR product size are summarized in Table 3, sourced from the NCBI primer blast database (https://www.ncbi.nlm.nih.gov/tools/primer-blast/).

### Histopathological analysis

Heart and aorta tissue samples preserved in formalin were subjected to paraffin embedding, sectioned at 5 µm, and stained with hematoxylin and eosin (H&E) stain according to standard protocols (28). Stained sections were examined under a light microscope, and photomicrographs were captured using ToupView software. 

### Statistical analysis

The Analysis of Variance (ANOVA) and Tukey’s honestly significant difference (Tukey’s HSD) test were used for statistical analysis through Minitab, and graphical representation was done through GraphPad Prism. A cluster heatmap of gene expression patterns was designed by using ChiPlot.

## Results

### Phytochemical analysis and anti-oxidant activity of FCLE

The FCLE contains a substantial number of flavonoids and phenolic compounds, which contribute significantly to its anti-oxidant activity, as evidenced by the DPPH and FRAP assays, as shown in Table 4. 

### Major bioactive compounds identified by HPLC

**Figure 1:**
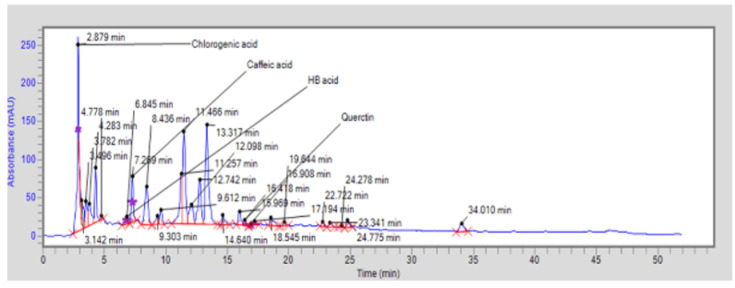
HPLC chromatogram of FCLE reveals the presence of key bioactive components such as chlorogenic acid, hydroxybenzoic acid, caffeic acid, and quercetin, which contribute to the extract’s antioxidant and therapeutic properties

**Figure 2:**
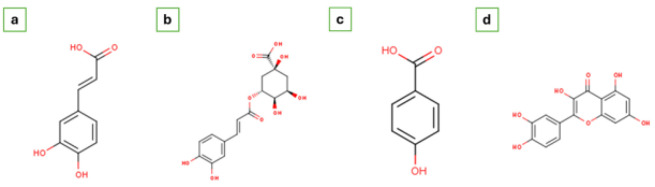
The 2-dimensional (2D) chemical structures of bioactive phytochemical constituents of FCLE (a). Caffeic acid (C_9_H_8_O_4_) PubChem ID: 689043 (b). Chlorogenic acid (C_16_H_18_O_9_) PubChem ID: 1794427 (c). Hydroxybenzoic acid (C_7_H_6_O_3_) PubChem ID: 135 (d). Quercetin (C_15_H_10_O_7_) PubChem ID: 5280343

HPLC analysis of FCLE revealed four bioactive compounds, illustrated in Figure 1. The identified compounds, chlorogenic acid, hydroxybenzoic acid, caffeic acid, and quercetin, are presented in Table 5 with their peak, retention time, area, height, and molecular formula. The compounds’ 2D chemical structures are displayed in Figure 2.

### Analysis of drug-likeness of FCLE phytochemicals

Physicochemical properties are crucial in determining the pharmacodynamics and pharmacokinetics of bioactive compounds. All bioactive phytochemical compounds of FCLE met Veber’s rule and Lipinski’s rule criteria, except chlorogenic acid, which violated one rule, as shown in Table 6. The identified phytochemical compounds from FCLE showed favorable drug-likeness and physicochemical properties that underscore their potential as therapeutic agents. Relevant ADME properties that include molecular weight (MW), topological polar surface area (TPSA), water solubility, octanol-water partition coefficients (Log P), gastrointestinal absorption (GIA), and blood-brain barrier (BBB) permeability are summarized in Table 7. Most of the bioactive phytochemicals from FCLE demonstrated acceptable TPSA values and GIA, with many crossing the BBB. Permeability glycoprotein (P-gp) influences the ADMET characteristics of various xenobiotics, limiting their cellular uptake and metabolism. None of the FCLE bioactive phytochemical compounds acted as the substrates of P-glycoprotein, showcasing their favorable profiles as potential therapeutic agents, as shown in Table 8. Cytochrome P450s (CYPs) are a substantial class of heme-containing enzymes essential for detoxifying foreign substances and metabolizing drugs, as illustrated in Table 8.

### Molecular docking

**Figure 3:**
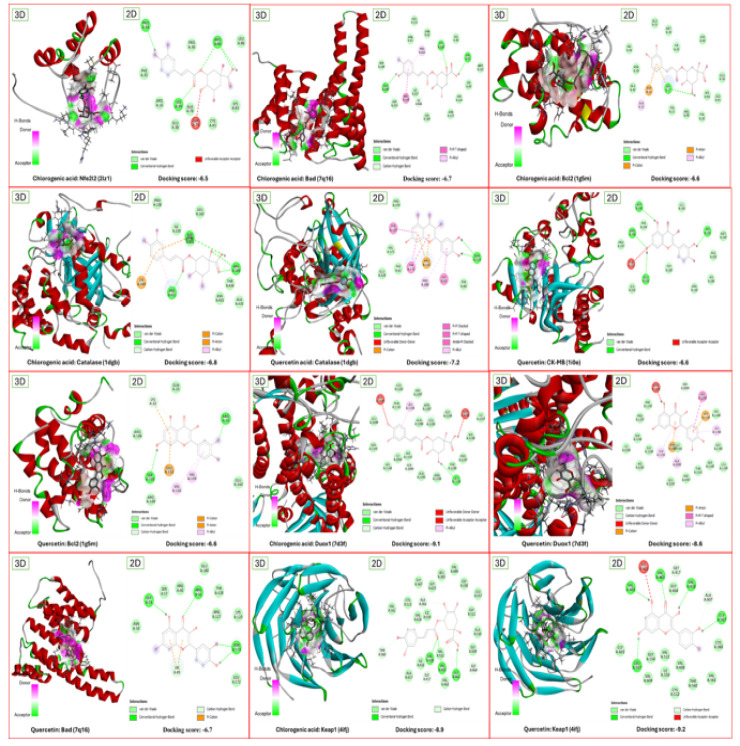
The 3-dimensional (3D) and 2-dimensional (2D) views of selected FCLE phytochemical compounds interacting with the target receptors

The docking score/binding affinity energies and the interacting amino acid residues for selected bioactive phytochemical compounds with their target receptors can be found in Table 9. The 3-dimensional (3D) and 2-dimensional (2D) representations of interactions between FCLE bioactive phytochemical compounds with their target receptors are depicted in Figure 3. According to molecular docking analysis, the bioactive phytochemical compounds chlorogenic acid and quercetin exhibited high affinity for target receptors Duox1 and Keap1 with the lowest binding energies of -8.5 kcal/mol.

### Biochemical parameters

#### Cardiac biomarkers (Troponin I, CK-MB)

**Figure 4:**
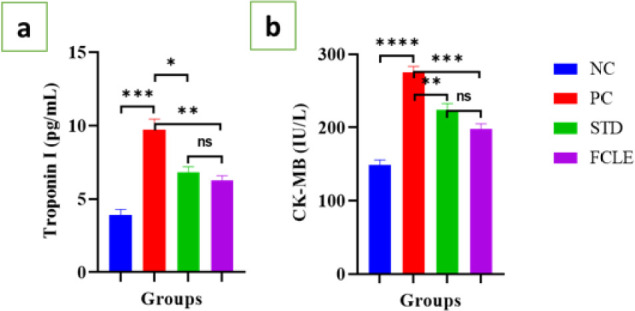
Graphs showing the changes in serum level of (a). Troponin I (pg/ml) and (b). CK-MB (IU/l) in different groups

The cardiac biomarkers Troponin I and creatinine kinase-MB (CK-MB), which rise in blood after MI, showed a significant (*P*<0.001) increase in the PC group compared to the NC group. STD treatment led to a significant reduction in serum troponin-I (*P*<0.05) and CK-MB (*P*<0.01) levels compared to the PC group. Similarly, the FCLE treatment group showed significantly reduced levels of Troponin I (*P*<0.01) and CK-MB (*P*<0.001) compared to the PC group, as illustrated in Figure 4. These findings suggest that FCLE possesses cardioprotective properties.

### Lipid profile (total cholesterol, triglycerides, HDL-C, and LDL-C)

**Figure 5:**
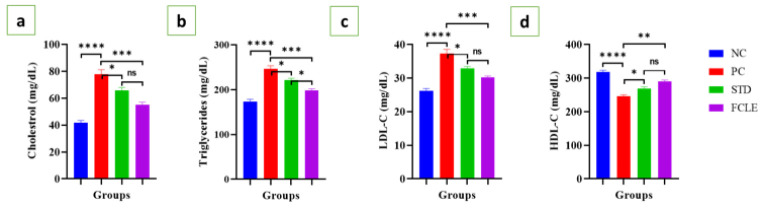
Graphs showing the changes in serum level of (a). Total cholesterol (mg/dl), (b). Triglycerides (mg/dl), (c). LDL Cholesterol (mg/dl), and (d). HDL cholesterol (mg/dl) in different groups. Results are presented as Mean±SEM. Statistical significance is denoted at a threshold of (*P*<0.05). **** P<0.0001, *** *P*<0.001, ** *P*<0.01, * *P*<0.05.

STD: Standard; NC: Negative control; PC: Positive control; FCLE: *Ficus carica* leaf extract

The levels of total cholesterol, triglycerides, and LDL-C significantly (*P*<0.0001) increased in the PC compared to the NC, while the HDL-C level significantly (*P*<0.0001) decreased compared to the NC group. STD led to a significant (*P*<0.05) decline in total cholesterol, triglycerides, and LDL-C levels, and a significant (*P*<0.05) increase in HDL-C. Similarly, FCLE also significantly (*P*<0.001) reduced total cholesterol, triglycerides, and LDL-C levels, while significantly (*P*<0.01) increasing HDL-C levels compared to the PC, as illustrated in Figure 5, indicating its potential to improve lipid metabolism and mitigate the risk of cardiovascular disease.

### Oxidative stress markers (SOD, CAT, MDA, NO)

**Figure 6:**
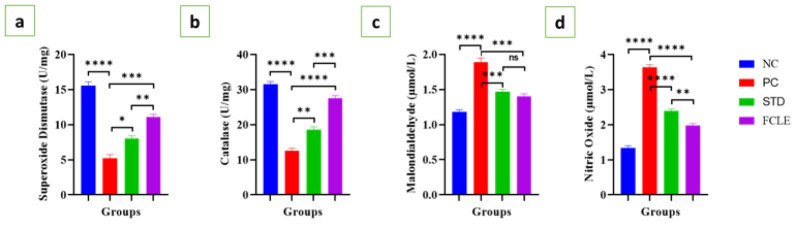
Graphs showing the changes in serum level of (a). Superoxide dismutase (U/mg), (b). Catalase (U/mg) (c). Malondialdehyde (μmol/l) and (d). NO (μmol/l) in different groups. Results are presented as Mean±SEM. Statistical significance is denoted at a threshold of (*P*<0.05). **** *P*<0.0001, *** *P*<0.001, ** *P*<0.01, * *P*<0.05.

STD: Standard; NC: Negative control; PC: Positive control; FCLE: *Ficus carica* leaf extract

The anti-oxidant enzymes superoxide dismutase (SOD) and catalase (CAT) demonstrated significantly (*P*<0.0001) decreased levels, indicating oxidative stress in PC compared to NC. The lipid peroxidation marker malondialdehyde (MDA) and nitric oxide (NO) levels were also significantly (*P*<0.0001) elevated in PC compared to the NC group. The STD group showed significantly increased SOD (*P*<0.05) and CAT (*P*<0.01) levels and significantly reduced MDA (*P*<0.001) and NO (*P*<0.0001) levels compared to the PC. Similarly, the FCLE group exhibited remarkably reduced oxidative stress, with significantly increased SOD (*P*<0.001) and CAT (*P*<0.0001), while significant declines in MDA (*P*<0.001) and NO (*P*<0.0001) levels were observed, as presented in Figure 6. Our findings suggest that the FCLE extract possesses potent anti-oxidant properties, which may contribute to its observed cardioprotective effects.

### Histopathological analysis

**Figure 7:**
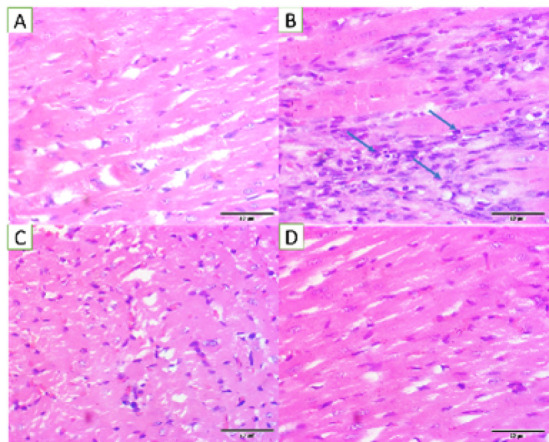
Photomicrographs of H&E-stained heart tissue at 40X (20 µm scale bar) sections from (A). NC group exhibited the characteristics of normal cardiomyocytic structures, with intact cells and fibrin bands (B). In the PC group, immune cell infiltration was visible with unclear cellular boundaries and abrupt myocardial disruptions (C). In the STD group, metoprolol treatment exhibited a positive effect on cardiomyocyte architecture, with recovery of dispersed cellular boundaries (D). FCLE group exhibited antioxidant and anti-inflammatory effects, where the immune infiltrate was seen to be cleared, and recovered cardiomyocytic structures

STD: Standard; NC: Negative control; PC: Positive control; FCLE: *Ficus carica* leaf extract

The NC group exhibited the characteristics of normal cardiomyocytic structures, with intact cells and fibrin bands. The intercalation among multiple cardiomyocytes and distinct morphological foci was visible (Figure 7A). In PC, ISO acted as a beta-agonist in mediating cardiac injury. Immune infiltration was visible with indistinct cellular boundaries and abrupt myocardial disruptions due to lipid peroxidation of fat deposits, due to HFD (Figure 7B). In the STD group, metoprolol treatment exhibited a positive effect on cardiomyocyte architecture, with the recovery of dispersed cellular boundaries. Oxidative stress-mediated microscopic foci were still apparent in the histology sections (Figure 7C). FCLE exhibited anti-oxidant and anti-inflammatory effects in mitigating ROS-mediated cellular damage, where immune infiltrates were seen to be cleared, and recovered cardiomyocytic structures were evident (Figure 7D). 

**Figure 8:**
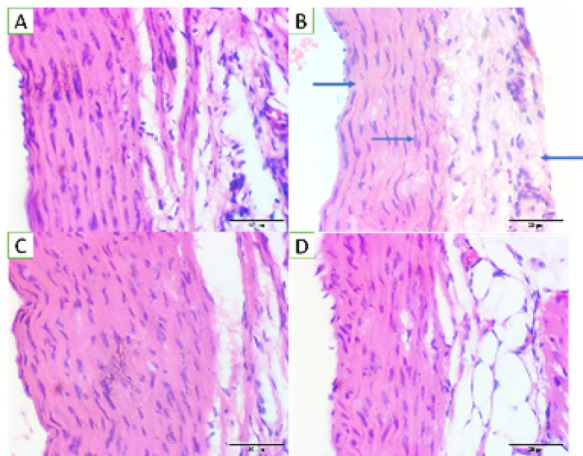
Photomicrographs of H&E-stained aorta at 40X (20 µm scale bar) sections from (A). Normal adventitia and tunica media were observed in the NC, where no tissue liquefaction occurred, and cellular boundaries were intact (B). In the PC, the liquefaction of the membranous layer in Tunica media and Tunica externa shows extensive damage (C). The STD recovered the injury, but still the damage persisted, and a slight liquefaction of the Tunica externa was seen (D). Relapses of thinned musculature in the tunica externa and tunica media to normal width and substantial cellular recovery in the adventitia were seen, with minimal damage to the musculature and immune infiltration in the FCLE group.

STD: Standard; NC: Negative control; PC: Positive control; FCLE: *Ficus carica* leaf extract

Normal adventitia and tunica media were observed in NC, where no tissue liquefaction occurred, and cellular boundaries were intact. The vasculature and cellular structures were visible, and nuclear material was condensed in the cell (Figure 8A). In the PC, the liquefaction of the membranous layer in Tunica media and Tunica externa explains extensive ROS-mediated damage when ISO was administered along with HFD. (Figure 8B). The STD treatment of metoprolol, being a beta-blocker, inhibited the activity of ISO in turbulent blood flow through the aortic vasculature, reducing the cardiac injury episodes. Still, after treatment, the induction pathway damage persisted, and a slight liquefaction of the tunica externa was seen (Figure 8C). Relapses of thinned musculature in the tunica externa and tunica media to normal width and substantial cellular recovery in the adventitia were seen, with minimal ROS-mediated damage of the musculature and immune infiltration, which explains the anti-oxidant potential of FCLE (Figure 8D).

### Molecular analysis

*Gene expression analysis of dual oxidases (Duox1, Duoxa1, and Duoxa2)*

**Figure 9:**
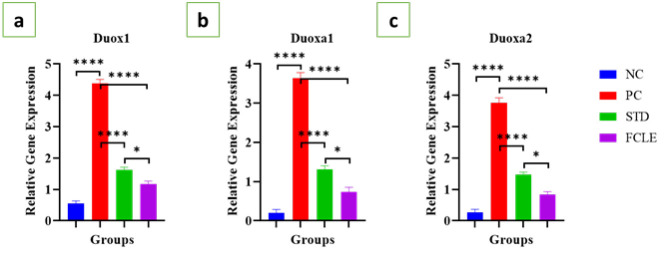
Graphs showing the relative gene expression level of (a) Duox1, (b) Duoxa1, and (c) Duoxa2 in different groups. Results are presented as Mean±SEM. Statistical significance is denoted at a threshold of (*P*<0.05). **** *P*<0.0001, *** *P*<0.001, ** *P*<0.01, * *P*<0.05. STD: Standard; NC: Negative control; PC: Positive control; FCLE: *Ficus carica* leaf extract

In the PC group, cardiac injury induced by ISO and HFD resulted in a significant (*P*<0.0001) up-regulation of Duox1, Duoxa1, and Duoxa2 gene expression compared to the NC, indicating heightened oxidative stress and inflammation. The STD group exhibited a significant (*P*<0.0001) decrease in the expression levels of Duox1, Duoxa1, and Duoxa2 compared to PC. The FCLE treatment group exhibited significant (*P*<0.0001) down-regulation of Duox1, Duoxa1, and Duoxa2 gene expression compared with the PC, as shown in Figure 9. Our findings suggest that FCLE may attenuate oxidative stress and inflammation by inhibiting the activation of the dual oxidase (DUOX) system.

*Nrf-2 signaling pathway (Nfe2l2, Nrf1 and Keap1)*

**Figure 10:**
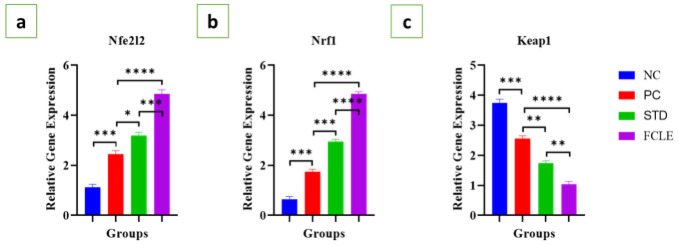
Graphs showing the relative gene expression level of (a) Nfe212, (b) Nrf1, and (c) Keap1 in different groups. Results are presented as Mean±SEM. Statistical significance is denoted at a threshold of (*P*<0.05). **** *P*<0.0001, *** *P*<0.001, ** *P*<0.01, * *P*<0.05. STD: Standard; NC: Negative control; PC: Positive control; FCLE: *Ficus carica* leaf extract

The PC exhibited significant (*P*<0.001) up-regulation of Nfe2l2 and Nrf1 gene expression compared to the NC, with a significant (*P*<0.001) decrease in Keap1 gene expression, indicating a compromised anti-oxidant response, as the observed changes, particularly the up-regulation of Nfe2l2, can be indicative of cellular stress and an attempt to compensate for increased oxidative damage. STD showed a significant rise in Nfe2l2 (*P*<0.05) and Nrf1 (*P*<0.001) gene expression and a significant (*P*<0.01) decrease in Keap1 gene expression compared to PC. Similarly, the FCLE treatment group exhibited a significant (*P*<0.0001) up-regulation of Nfe2l2 and Nrf1 gene expression and a significant (*P*<0.0001) down-regulation of Keap1 gene expression compared to the PC, as shown in Figure 10. Our findings suggest that FCLE has the potential to modulate the Nrf2/Keap1 pathway and enhance anti-oxidant defense mechanisms.

*Gene expression analysis of apoptosis-related genes*

**Figure 11:**
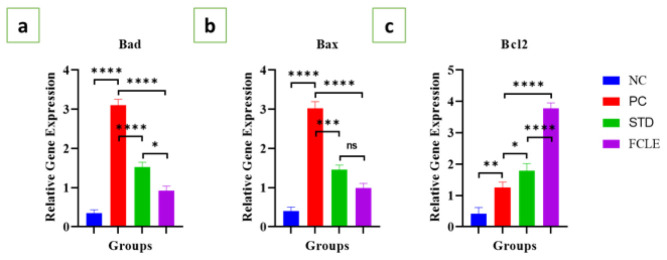
Graphs showing the relative gene expression level of (a) Bad, (b) Bax, and (c) Bcl2 in different groups. Results are presented as Mean±SEM. Statistical significance is denoted at a threshold of (*P*<0.05). **** *P*<0.0001, *** *P*<0.001, ** *P*<0.01, * *P*<0.05. STD: Standard; NC: Negative control; PC: Positive control; FCLE: *Ficus carica* leaf extract

In the PC, there was significant (*P*<0.0001) up-regulation of pro-apoptotic genes Bad and Bax, and a significant (*P*<0.01) increase in Bcl2 gene expression compared to the NC group. Our findings indicate increased apoptotic cell death, with the heart likely attempting to compensate for the injury by up-regulating the survival factor Bcl-2. The STD group showed a significant decline in Bad (*P*<0.0001) and Bax (*P*<0.001) expression and a significant (*P*<0.05) increase in Bcl2 gene expression compared to the PC. The FCLE treatment group exhibited substantial (*P*<0.0001) down-regulation of Bad and Bax, along with significant (*P*<0.0001) up-regulation of Bcl2 gene expression compared to the PC, as shown in Figure 11. Our results suggest that the FCLE extract inhibits apoptosis and promotes cell survival. 

**Figure 12:**
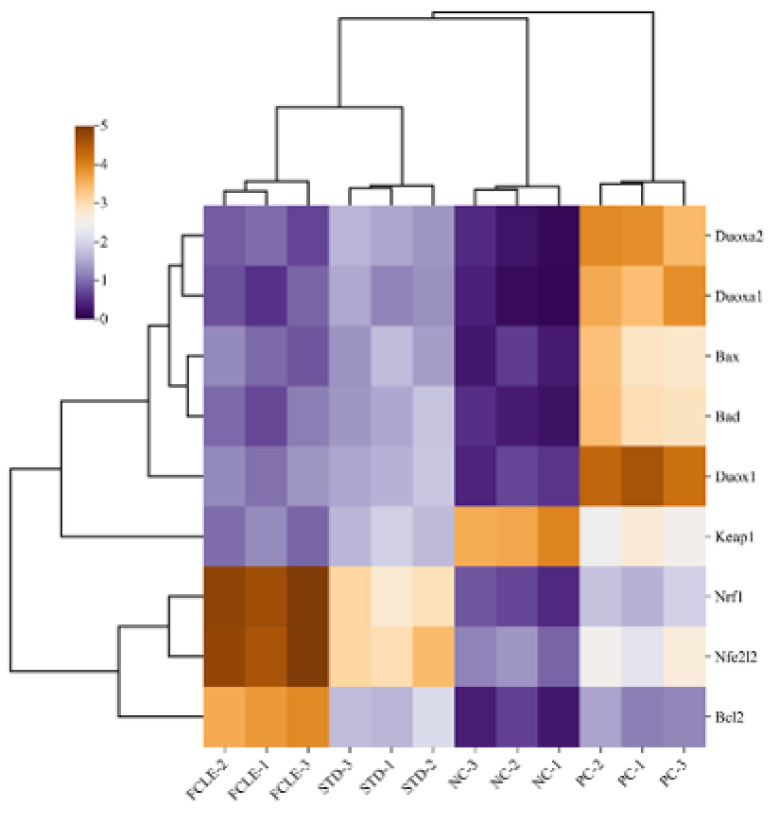
Cluster heatmap of relative gene expression pattern across the different treatment groups of dual oxidases (Duox1, Duoxa1, and Duoxa2), Nrf-2 signaling pathway (Nfe2l2, Nrf1, and Keap1), and apoptosis-related genes (Bax, Bad, and Bcl2)

**Figure 13:**
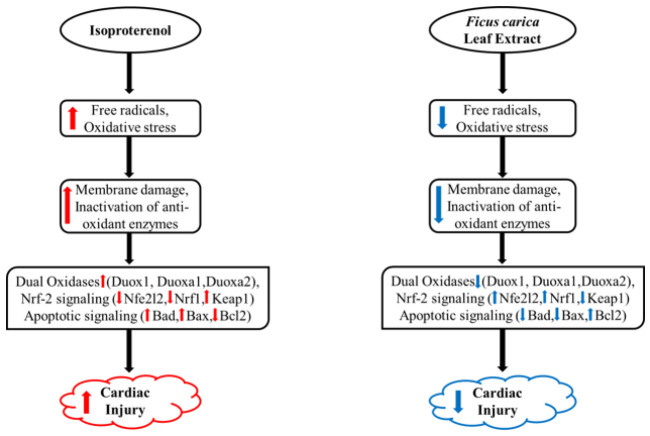
The left side shows the mechanism of isoproterenol-induced myocardial infarction, and the right side of the figure exhibits the cardio-protective mechanism of FCLE extract to ameliorate myocardial infarction through key signaling pathways involved in oxidative stress, inflammation, and apoptosis, including dual oxidases, Nrf2/Keap1 pathway, and related apoptotic signaling cascades

To visualize gene expression patterns across the different treatment groups of dual oxidases (Duox1, Duoxa1, and Duoxa2), Nrf-2 signaling pathway (Nfe2l2, Nrf1, and Keap1), and apoptosis-related genes (Bax, Bad, and Bcl2), hierarchical clustering was performed, and results were illustrated in Figure 12. The cardioprotective mechanism of FCLE in ameliorating MI through key signaling pathways involved in oxidative stress, inflammation, and apoptosis, including dual oxidases, the Nrf2/Keap1 pathway, and associated apoptotic signaling cascades, is exhibited in Figure 13.

## Discussion

During MI, an elevated level of ROS overwhelms the body’s natural defenses, resulting in oxidative stress that harms cardiomyocytes (29, 30). The anti-oxidant properties of FCLE, due to the presence of flavonoids and phenolic compounds, help mitigate oxidative stress by reducing free radical formation, boosting anti-oxidant defenses, and reducing myocardial injury (19, 31). FCLE can enhance anti-oxidant enzyme activity while mitigating lipid peroxidation and protein oxidation (32). FCLE increases the activity of SOD, which is essential for transforming superoxide radicals into hydrogen peroxide (33), along with increased catalase activity, which further detoxifies hydrogen peroxide produced by SOD activity (34), indicating the FCLE potential as a natural anti-oxidant agent. The auto-oxidation of ISO creates a significant amount of ROS that targets polyunsaturated fatty acids in cell membranes, leading to the formation of peroxyl radicals that subsequently attack nearby fatty acids in cell membranes, initiating a chain reaction that causes lipid peroxidation (35). Administration of FCLE has been associated with notable reductions in MDA levels, an indicator of lipid peroxidation (34). The elevated levels of nitric oxide in the PC group likely reflect excessive inducible nitric oxide synthase (iNOS) activation due to oxidative/nitrosative stress (36). The FCLE group indicates attenuation of excessive NO production due to its anti-oxidant potential (37). The research reported that FCLE significantly decreases the serum levels of troponin I and CK-MB, critical markers of MI (38). Metoprolol, used as standard treatment in our study, exerted beneficial effects on lipid metabolism by decreasing triglycerides, LDL cholesterol, and total cholesterol. However, the increase in HDL cholesterol in our study is contraindicated by the previous study, which may be due to the duration of metoprolol administration (39). FCLE improves lipid profiles with marked reductions in LDL cholesterol and triglycerides, indicating an overall enhancement in the lipid profile (40, 41).

NADPH oxidases are part of the flavoenzyme family, which includes DUOX and NOX proteins linked to ROS production in various tissues and cells (42). In the heart, NOX 2 and NOX 4 are the primary sources of superoxide (O_2_^-^) and (H_2_O_2_), contributing to ROS production and subsequent cardiac injury (43). The up-regulation of DUOX can lead to chronic oxidative stress and cardiac fibrosis (44). The bioactive compounds present in FCLE significantly influence the gene expression levels of DUOX due to their anti-oxidant activity (45). The Nrf2/Keap1 pathway is crucial for regulating cytoprotective response against ROS-generated oxidative stress and acts as a key signaling pathway for reducing the size of infarction (46, 47). Under normal conditions, Keap1 inhibits Nrf2 by binding to it, which shortens the half-life of Nrf2 by promoting its degradation in the proteasome. However, Keap1 undergoes structural changes during oxidative stress and prevents Nrf2 from binding and translocating into the nucleus (46, 48). The genetic activation of Nrf2 via Keap1 knockdown suppresses the onset of disease, which is evidence of Nrf2 activity (49). Our study reported that FCLE boosts the expression of the Nfe-212 gene and down-regulates the Keap-1 expression due to its anti-oxidant potential by facilitating the dissociation of Nfe2l2 from Keap-1, resulting in its activation (44, 50). The bioactive compounds in FCLE carry cardioprotective effects that modulate apoptotic pathways, thereby favoring cell survival (51, 52).

The ADMET properties of bioactive phytochemical compounds are essential for successful drug development (53). Drug candidates are often poorly absorbed when their topological polar surface area (TPSA) exceeds 140 Å2, which serves as a benchmark for marketed drugs. TPSA shows a positive correlation with mass, where molecules exceeding 500 g/mol generally have TPSA values above 140 Å² (54). TPSA values and GIA for many bioactive phytochemical compounds in FCLE were found to be acceptable. Log p-value and Abbott bioavailability scores above zero suggest that these phytochemicals exhibit notable bioavailability and effectively pass through the cell membrane (55). GIA and the permeability of the BBB are vital features for drugs intended for broad application (56). Most of the bioactive phytochemical compounds from FCLE displayed acceptable GIA and were able to cross the BBB. Permeability glycoprotein (P-gp) influences the ADMET characteristics of many xenobiotics, restricting cellular uptake and metabolism by functioning as a one-way efflux pump, expelling substrates from inside cells to the exterior (57). None of the bioactive phytochemical compounds in FCLE were substrates of P-glycoprotein, indicating their desirable characteristics as potential therapeutic agents.

**Table 1:** Shows the four groups of Wistar rats (n=8 per group) and their treatments to investigate cardiac injury

Group name	Treatment
Negative control (NC)	Basic chow diet + Water *ad libitum*
Positive control (PC)	High-fat diet for 14 days + Isoproterenol 30mg/kg/day subcutaneously on 13^th^ and 14^th^ day
Standard (STD)	High-fat diet for 14 days + Isoproterenol 30 mg/kg/day subcutaneously on 13^th^ and 14^th^ day + Metoprolol 20mg/kg/day orally from day 14 to 21
FCLE treatment (FCLE)	High-fat diet for 14 days + Isoproterenol 30mg/kg/day subcutaneously on 13^th^ and 14^th^ day + *Ficus carica* leaf extract 300 mg/kg/day orally from day 14 to 21

**Table 2:** Composition of basic chow diet (BCD) per Kg of weight used as normal diet, and Dalda ghee per 100 g of weight used to prepare a high-fat diet

Component	BCD (per Kg)	Ghee (per 100 g)
Maize	620 g	-
Soybean meal	180 g	-
Full-fat soybean	130.5 g	-
Wheat offal	40 g	-
Bone meal	20.5 g	-
Lysine	2 g	-
Methionine	3 g	-
Salt	4 g	-
Moisture	~100 g	-
Total fat	20 g	100 g
Saturated fat	-	45 g
Monounsaturated fat	-	40.5 g
Polyunsaturated fat	-	14.5 g
Trans fat	-	<0.5 g
Protein	220 g	0 g
Fiber	60 g	0 g
Ash	80 g	-
Cholesterol	0 g	0 g
Energy	2900 kcal/Kg	900 kcal/100 g
Vitamin A	-	4900 IU
Vitamin D	-	490 IU

**Table 3:** Primers used for relative gene expression analysis with their names, rat genome database ID (RGD ID), accession number, type, 5’-3’ oligonucleotide sequence, GC content %, annealing temperature, and product length

Gene	RGD ID	NCBI Gene ID/accession number	Type	Oligonucleotide sequence (5'-3')	GC%	Annealing temperature	Product length
β-actin	628837	NM_031144.3	Forward	CCGCGAGTACAACCTTCTTG	55.00	58.93	71
			Reverse	CGTCATCCATGGCGAACTGG	60.00	61.43	
Duox1	628760	NM_153739.3	Forward	CGAAGACAGCGTCATCCCC	63.16	60.52	71
			Reverse	CCGTCGAATCTCTTGAGGAGG	57.14	59.93	
Duoxa1	1306601	NM_001107767.1	Forward	AGCAGGGAGACACAACCTAC	55.00	59.02	89
			Reverse	AACAGTTTATGGGCCTGTCGG	52.38	60.61	
Duoxa2	1560628	NM_001191965.2	Forward	GGGGTGCTACCCTTTTACCC	60.00	60.03	71
			Reverse	AGGATAACGATGAGCAGCGG	55.00	59.97	
Nfe2l2	620360	XM_063284651.1	Forward	TGCTCCGACTAGCCATTGAC	55.00	59.82	92
			Reverse	CATCATGCTGAGGGCGGAC	63.16	60.89	
Nrf1	1304603	NM_001100708.1	Forward	CCGTTGGAGCACTTACTGGA	55.00	59.68	78
			Reverse	TCACGGCTTTGCTGATGGTC	55.00	60.96	
Keap1	621619	NM_057152.2	Forward	ATCTAGGGCATCCTGCTCCC	60.00	60.84	82
			Reverse	ACAGAAGTTGGGTCATTGGCT	47.62	59.85	
Bax	2192	NM_017059.2	Forward	CTGGGATGGCCTCCTTTCC	63.16	59.77	75
			Reverse	GTGAGGACTCCAGCCACAAA	55.00	59.89	
Bad	620103	NM_022698.2	Forward	CTTGAGGAAGTCCGATCCCG	60.00	59.90	82
			Reverse	CATACTCTGGGCTGCTGGTC	60.00	60.18	
Bcl2	2199	NM_016993.2	Forward	GAACTGGGGGAGGATTGTGG	60.00	60.03	80
			Reverse	GGGGTGACATCTCCCTGTTG	60.00	60.04	

**Table 4:** Showing the assays for phytochemical analysis (TPC and TFC) and anti-oxidant activity assays (DPPH and FRAP) of *Ficus carica* leaf extract, indicating the concentration of phytochemicals and their anti-oxidant power

Assays	Mean ± SEM values
TPC (mg GAE/g)	437.38 ± 0.86
TFC (mg CE/g)	24.16 ± 0.79
DPPH (% Inhibition)	50.59 ± 0.34
FRAP (mmol Fe^2+^ Eq/mg extract)	5.86 ± 0.09

DPPH: 2,2-Diphenyl-1-picrylhydrazyl, TPC: Total phenolic content, FRAP: Ferric reducing anti-oxidant power, TFC: Total flavonoid content

HPLC: High-performance liquid chromatography, FCLE: *Ficus carica* leaf extract

**Table 5:** HPLC of FCLE showing bioactive phytochemical compounds and their molecular formula, retention time, area, and height

Peak	Name of the compound	Molecular formula	Retention time	Area	Height
1	Chlorogenic acid	C_16_H_18_O_9_	2.879	2497800.6	227540.5
2	Hydroxybenzoic acid	C_7_H_6_O_3_	6.845	177977.2	9071.9
3	Caffeic acid	C_9_H_8_O_4_	7.269	683961.4	60001.6
4	Quercetin	C_15_H_10_O_7_	16.418	121644.0	7855.8

HPLC: High-performance liquid chromatography, FCLE: *Ficus carica* leaf extract

FCLE: *Ficus carica* leaf extract

**Table 6:** Drug-likeness analysis and phytochemical properties of FCLE bioactive phytochemical compounds, underscoring their potential as therapeutic agents

Compound	MW (g/mol)	Lipophilicity (log p)	HBD	HBA	TPSA (Å2)	ROTB	LRV	VRV
Quercetin	302.24	1.23	5	7	131.36	1	0	0
Chlorogenic acid	354.31	-0.39	6	9	164.75	5	1	1
Caffeic acid	180.16	0.93	3	4	77.76	2	0	0
Hydroxybenzoic acid	138.12	1.05	2	3	57.53	1	0	0

MW: Molecular weight, log p: octanol-water partition coefficients, TPSA: Topological polar surface area, ROTB: Number of rotatable bonds, LRV: Lipinski’s rule violations, VRV: Veber’s rule violations

**Table 7:** Physicochemical properties of FCLE bioactive phytochemical compounds showing acceptable water solubility, GIA, along with many crossing the BBB

Compound	Water solubility	GIA	BBB permeant	ABS
Quercetin	Soluble	High	No	0.55
Chlorogenic acid	Soluble	Low	No	0.11
Caffeic acid	Soluble	High	No	0.56
Hydroxybenzoic acid	Soluble	High	Yes	0.85

GIA: Gastrointestinal absorption, BBB: Blood-brain barrier, ABS: Abbott’s bioavailability score; FCLE: *Ficus carica* leaf extract

**Table 8:** The interaction of FCLE bioactive phytochemical compounds with P-glycoprotein and cytochrome P450 isoenzymes showcases their favorable profiles as potential therapeutic agents

Compound	Pgp substrate	CYP1A2 inhibitor	CYP2C19 inhibitor	CYP2C9 inhibitor	CYP2D6 inhibitor	CYP3A4 inhibitor
Quercetin	No	Yes	No	No	Yes	Yes
Chlorogenic acid	No	No	No	No	No	No
Caffeic acid	No	No	No	No	No	No
Hydroxybenzoic acid	No	No	No	No	No	No

Pgp: P glycoprotein, CYP1A2: Cytochrome P450 1A2, CYP2C19: Cytochrome P450 2C19, CYP2C9: Cytochrome P450 2C9, CYP2D6: Cytochrome P450 2D6, CYP3A4: Cytochrome P450 3A4; FCLE: *Ficus carica* leaf extract

**Table 9:** The binding affinity energies/docking score of FCLE (Ficus carica leaf extract) selected bioactive phytochemical compounds with their target receptors, along with interacting amino acid residues, support the cardioprotective potential of FCLE

Phytochemicals (CID)	Target (PDB ID)	Docking score (kcal/mol)	Interacting amino acid residues
Chlorogenic acid (1794427)	Catalase (1dgb)	-6.8	PRO158, LEU160, ILE159, ASP157, PHE:432, ARG189, LYS349, ARG431, THR434, ASN433, ALA435
	Duox1 (7d3f)	-9.1	LEU1202, PHE1097, GLY1195, THR1141, LEU1198, ARG1087, GLY1239, SER1240, ILE1237, LEU1199, ALA1090, LEU1235, HIS1238, SER1145, HIS1148, ILE1093, HIS1144, SER1094, ALA1091, ARG1248, PHE1249, PRO1247, LEU1246
	Nfe2l2 (2lz1)	-6.5	PRO34, PRO36, ARG84, LEU86, PHE35, ARG16, LYS39, ALA78, LYS83, GLU38, GLN79, CYS81
	Keap1 (4ifj)	-8.9	VAL604, LEU365, GLY558, GLY367, GLY605, VAL561, CYS513, VAL606, ALA366, ILE559, LEU557, GLY511, ALA510, THR560, ILE416, VAL418, VAL512, VAL465, GLY509, GLY464, ALA607, GLY417, VAL463, GLY462
	Bad (7q16)	-6.7	GLU113, ARG41, ASN42, PRO165, LYS120, VAL46, SER45, MET121, SER210, GLU209, ASP213, HIS164, ILF217, ILE166, LYS49, ASP124, GLY169, ASN173
	Bcl2 (1g5m)	-6.6	GLU13, MET16, VAL36, HIS20, SER49, ASN39, GLU42, ASP31, LYS17, HIS94, GLU50, LEU95, ALA32, TYR21, ARG98, TYR18
Quercetin (5280343)	CK-MB (1i0e)	-6.6	ASP195, LEU193, ARG236, PHE194, HIS191, MET240, PRO197, GLU231, GLU232, TRP228, PHE192, HIS296, LEU202, ARG130
	Catalase (1dgb)	-7.2	PRO374, TYR137, VAL375, GLU328, PHE82, TYR379, ARG320, PRO108, GLY83, ASN319, TYR84
	Duox1 (7d3f)	-8.6	ARG1248, SER1094, PHE1249, PRO1247, HIS1238, HIS1144, HIS1148, LEU1198, GLY1195, SER1240, ILE1236, GLY1239, LEU1235, ARG1087, ILE1093, ALA1090, SER1145, LEU1199, HIS1250, THR1141, PHE1097, LEU1202
	Keap1 (4ifj)	-9.2	ILE416, VAL463, VAL465, GLY464, GLY417, VAL418, ALA607, GLY367, GLY603, LEU557, GLY558, VAL512, ILE559, VAL606, VAL604, THR560, CYS368, VAL561, CYS513
	Bad (7q16)	-7.1	GLU180, GLY53, SER57, ARG60, ARG56, TYR128, ARG127, LYS120, ASN50, ASN173, LYS49, LEU172
	Bcl2 (1g5m)	-6.6	GLN25, LYS22, ARG106, ARG26, SER105, GLU152, VAL159, VAL156, GLU160, ARG109

FCLE: *Ficus carica* leaf extract

Results are presented as Mean±SEM. Statistical significance is denoted at a threshold of (*P*<0.05). **** *P*<0.0001, *** *P*<0.001, ** *P*<0.01, * *P*<0.05.

STD: Standard; NC: Negative control; PC: Positive control; FCLE: *Ficus carica* leaf extract

In the heatmap, the rows represent genes, and the columns represent groups.

FCLE: *Ficus carica* leaf extract

## Conclusion

FCLE presents a promising natural therapeutic option for enhancing cardiac protection against oxidative stress and cardiac injury by modulating the key signaling pathways involved in oxidative stress, inflammation, and apoptosis, including dual oxidases, the Nrf2/Keap1 pathway, and related apoptotic signaling cascades, warranting further exploration as an adjunctive treatment in CVDs.

## Declaration

The authors declare that no artificial intelligence (AI) tools or technologies were used in the preparation, writing, data collection, analysis, or editing of this manuscript. All content is original and has been developed solely by the authors.
